# A Class-Selective Immunoassay for Sulfonamides Residue Detection in Milk Using a Superior Polyclonal Antibody with Broad Specificity and Highly Uniform Affinity

**DOI:** 10.3390/molecules24030443

**Published:** 2019-01-26

**Authors:** Chenglong Li, Xiangshu Luo, Yonghan Li, Huijuan Yang, Xiao Liang, Kai Wen, Yanxin Cao, Chao Li, Weiyu Wang, Weimin Shi, Suxia Zhang, Xuezhi Yu, Zhanhui Wang

**Affiliations:** 1 Beijing Advanced Innovation Center for Food Nutrition and Human Health, College of Veterinary Medicine, China Agricultural University, Beijing Key Laboratory of Detection Technology for Animal-Derived Food Safety, Beijing Laboratory of Food Quality and Safety, Beijing 100193, China; mylichenglong@cau.edu.cn (C.L.); lxscau@163.com (X.L.); huijuanyang@cau.edu.cn (H.Y.); wenkai@cau.edu.cn (K.W.); laura12390@163.com (Y.C.); chaolicau2018@gmail.com (C.L.); weiyuwang2018@gmail.com (W.W.); shiwm@cau.edu.cn (W.S.); suxia@cau.edu.cn (S.Z.); wangzhanhui@cau.edu.cn (Z.W.); 2 Henan Animal Health Supervision Institute, Zhengzhou 450008, China; liyonghan1020@126.com; 3 College of Veterinary Medicine, Qingdao Agricultural University, Qingdao 266109, China; liangxiao4000@163.com

**Keywords:** milk safety, multi-sulfonamides, novel hapten, polyclonal antibody, broad specificity, highly uniform affinity

## Abstract

The development of multianalyte immunoassays with an emphasis on food safety has attracted increasing interest, due to its high target throughput, short detection time, reduced sample consumption, and low overall cost. In this study, a superior polyclonal antibody (pAb) against sulfonamides (SAs) was raised by using a bioconjugate of bovine serum albumin with a rationally designed hapten 4-[(4-aminophenyl) sulfonyl-amino]-2-methoxybenzoic acid (SA10-X). The results showed that the pAb could recognize 19 SAs with 50% inhibition (IC_50_) below 100 µg L^−1^ and a recognition profile for SAs containing, either a five-atom ring or a six-atom ring, with highly uniform affinity. A three-dimensional quantitative structure-activity relationship analysis indicated that the electrostatic features of SAs play a considerably important role, during recognition with pAb than stereochemical effects. Skimmed milk samples were directly diluted five times before analysis. After optimization, the limit of detection for sulfamonomethoxine, sulfamethoxazole, sulfaquinoxaline, sulfadimethoxine, and sulfamethazine were 1.00, 1.25, 2.95, 3.35, and 6.10 µg L^−1^, respectively. The average recoveries for these 5 SAs were 72.0–107.5% with coefficients of variation less than 14.1%. The established method, based on pAb, with broad specificity and uniform affinity, offered a simple, sensitive, and high-throughput screening tool for the detection of multi-SAs in milk samples.

## 1. Introduction

Sulfonamides (SAs) are a group of synthetic chemotherapeutics with a common *p*-aminobenzenesulfonamide moiety. They are widely used in veterinary practice for the treatment of infectious diseases and utilized as growth-promoting feed additives [1]. The widespread use of SAs in animal husbandry, and non-compliance with withdrawal time, can enrich chemical residues in animal-derived foods. The presence of SAs residue in food can create potential human health hazards, due to allergic or toxic effects. Furthermore, the extensive use of SAs in animal stock husbandry has been associated with the multi-drug resistance in pathogens leading to worldwide concern [2,3]. Therefore, in most countries (including China, the United States of America, and the European Union) maximum residue limits (MRLs) have been established for SAs in food at 100 μg kg^−1^ [4,5,6]. 

Immunoassays are one of the most important and widely used rapid detection methods for food safety. They are simple, fast, sensitive, and specific [7]. However, structurally similar contaminants can be present in samples rather than the one defined chemical compound [8]. Thus, to simultaneously determine a class of targets in a single test, multianalyte immunoassays have attracted significant attention. The key reagent in immunoassays is the antibody, and the quality of this antibody determines the immunoassay performance. For SAs with a common substructure, it is critical that the antibody recognizes all of them with broad specificity and uniform affinity. 

To achieve this goal, much effort has been made by different researchers. Muldoon et al. first produced a monoclonal antibody (mAb), Sulfa-1, that could recognize eight SAs with 50% inhibition (IC_50_) below 10 mg L^−1^ [9]. Then mAbs 27G3A9B10 and 4E10B12B6E12, that are prepared by Haasnoot et al., could recognize eight SAs with IC_50_ values below 100 µg L^−1^ [10,11]. Cliquet et al. developed an mAb 3B5B10E3 that could detect 5 SAs with IC_50_ values, ranging from 9–100 µg L^−1^ [12]. Zhou et al. later produced an mAb 4E5, that could cross-react with 16 SAs with IC_50_ values less than 100 µg L^−1^ [13]. Franek et al. and Korpimäki, et al. produced a polyclonal antibody (pAb), and recombinant antibody, respectively, that could simultaneously detect 17 and 18 SAs at the regulatory levels [14,15]. An mAb with IC_50_ values for 26 SAs below 100 µg L^−1^ was also described [16]. However, these antibodies have a diverse range of IC_50_ values for different SAs and are not desirable for class-specific detection of SAs with a non-uniform cross-reactivity (CR). 

To prepare antibodies with uniform recognition spectra, our group has developed mAbs (1D10, 4C7 and 4D11) against SAs [17]. The mAb 1D10 and 4C7 could recognize 10 and 13 SAs, respectively, with their IC_50_ values below 100 µg L^−1^. The mAb 4D11, derived from hapten SA10, could recognize 14 SAs with IC_50_ values of 1.2–36.8 µg L^−1^, within 1 order of magnitude. However, among these SAs, only sulfamethoxazole (SMX) had a five-atom-ring; the other 13 SAs were all containing a six-atom-ring. That is, mAb 4D11 has worse performance towards SAs containing a five-membered ring. Therefore, based on hapten SA10, a desirable hapten should be designed for the production of a superior antibody with broad specificity and highly uniform affinity. 

The aim of the study was to produce an antibody that could recognize, not only SAs containing a six-membered ring, but also SAs containing a five-membered ring with uniform IC_50_ values. The novel hapten SA10-X (referred to hapten SA10) was rationally designed. Based on the produced superior pAb, with broad specificity and highly uniform affinity, a class-specific multianalyte immunoassay was developed for SAs in milk samples. Furthermore, molecular modeling was used to get structural insights into the pAb recognition profiles. 

## 2. Results and Discussion 

### 2.1. Rational Design of Novel Hapten SA10-X 

**Figure 1:**
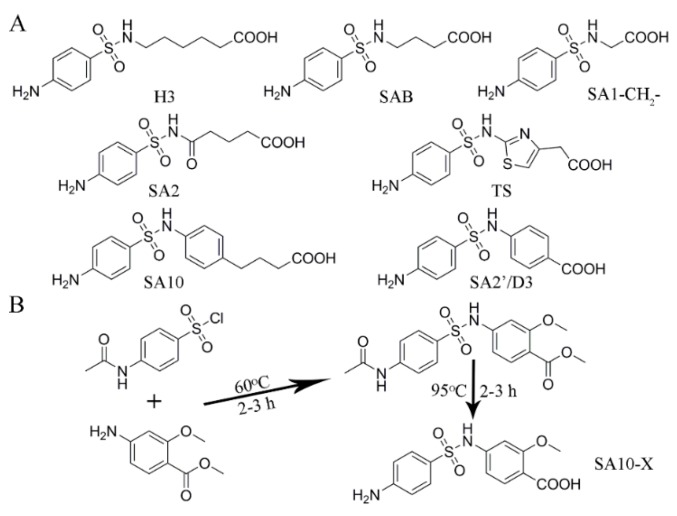
The chemical structures of published haptens discussed in this study (**A**). Hapten H3, SAB, SA1-CH_2_-, and SA2 represent the single-ring haptens, and TS, SA10 and SA2’ (D3) represent the two-ring haptens. (**B**) The synthesis of hapten SA10-X.

SAs are a class of synthetic antimicrobial agents that can be divided into single-ring SAs and two-ring SAs containing either a five-atom ring or a six-atom ring [18]. Due to the variation of the heterocyclic five/six-atom rings, the produced antibodies can rarely achieve the uniform recognition ability towards SAs, containing a five-atom rings and SAs containing a six-atom rings. In a prior study conducted in our laboratory, hapten H3 (Figure 1A) mimicking the common sub-structure of SAs without the heterocyclic five/six-atom ring, was used to produce an antibody. It is expected to elicit an antibody with a broad recognition spectrum [19]. However, the results showed that this pAb can only recognize 7 SAs with IC_50_ values below 100 mg L^−1^. Instead of the (CH_2_)_5_-spacer arm in H3, the hapten SAB with a (CH_2_)_3_-spacer arm (Figure 1A) was used to raise pAb [20]. Unfortunately, the resulting pAb had affinity towards 7 SAs with IC_50_ values that ranged from 1.6–88 µg L^−1^. Furthermore, hapten SA1-CH_2_ (Figure 1A), with the shortest spacer length, was employed to prepare an antibody, but only 10 SAs had IC_50_ values below 100 µg L^−1^ when using anti-SA1-CH_2_ pAb [14]. In addition, hapten SA2 with a -CO-(CH_2_)_3_ spacer (Figure 1A) was utilized, but the resulting pAb cannot detect any SAs under 100 µg L^−1^ [21]. Thus, haptens with only one single-ring aminobenzenesulfonylamino moiety, might not be suitable for group-specific antibody production. The heterocycle in the hapten plays an important role during the generation of group-specific antibody against SAs. 

Two-ring haptens, such as TS containing a five-membered thiazole ring (Figure 1A), were used in our previous study [17]. The mAb 4C7 generated from TS can bind to 13 SAs with IC_50_ values of 0.615–91.6 µg L^−1^. For two SAs (containing a six-atom ring) under surveillance, 4C7 with an IC_50_ value of 154.2, and 14231 µg L^−1^, for sulfamerazine (SMR), and sulfamethazine (SMZ), respectively definitely cannot be employed in practical use. Similar results were found in mAb 27G3, produced by hapten TS, which IC_50_ was 500 µg L^−1^ for SMR and 8000 µg L^−1^ for SMZ [10,11]. In contrast, mAb 4D11 raised from hapten SA10 (Figure 1A) with a Ph-(CH_2_)_3_ spacer arm had perfect recognition ability against SAs, containing a six-atom ring [17]. But for SAs containing a five-atom ring, the IC_50_ values ranged from 330.4–11419 µg L^−1^ except SMX with 6.6 µg L^−1^. The other pAb raised from SA2’, with a Ph-spacer arm (Figure 1A) had affinity for SAs containing a five-atom ring with IC_50_ values of 0.4–95.6 µg L^−1^, but 311 µg L^−1^ for SMZ and 426 µg L^−1^ for SDM’ [14]. In addition, hapten D3 (Figure 1A) with the same structure as SA2’ was utilized to generate pAb. The product showed an IC_50_ for STZ of 2 µg L^−1^, SMT of 12.8 µg L^−1^, and SDM’ of 31 µg L^−1^; however, SIZ, SMX, SXL (containing a five-membered ring), as well as SDM and SMZ (containing a six-membered ring) were all over 100 µg L^−1^ [22]. This analysis indicated hapten, with the common *p*-aminobenzenesulfonamide moiety, the second ring with stereochemical and electronic properties make it better for eliciting a generic immune response against two-ring SAs. 

Based on the hapten structures of SA10, SA2’, and D3, as well as their corresponding immune responses against SAs, a novel hapten SA10-X was designed by introducing a methoxy group on the benzene ring at the *meta-*position of SA2’ (Figure 1A). The methoxy group has stereochemical and electronic characters that expect to elicit antibodies with a broader binding pocket to combine SAs, containing either a five-atom ring or a six-atom ring with broad specificity and highly uniform affinity. 

### 2.2. Synthesis and Identification of Hapten SA10-X 

**Figure 2:**
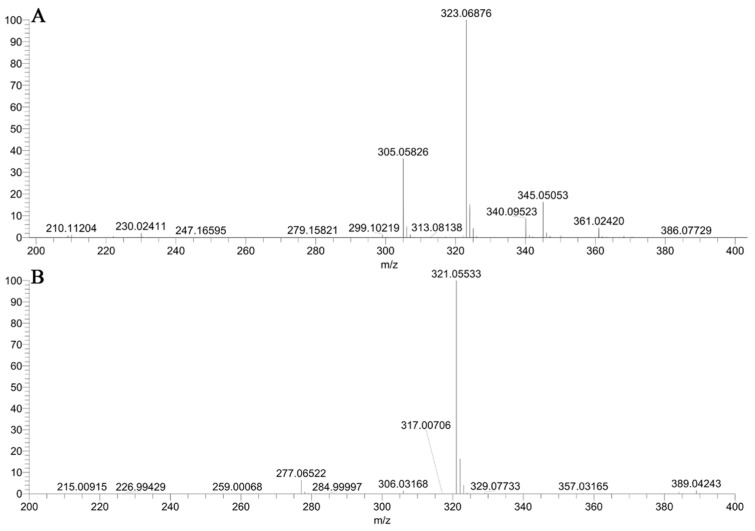
The MS spectra of hapten SA10-X acquisition under positive ionization mode (**A**) and negative ionization mode (**B**) using UHPLC-MS/MS.

Synthesis route for hapten SA10-X has been shown in Figure 1B. Detailed synthesis protocols are in the Method section. The synthesized SA10-X was identified by MS, ^1^H-NMR, and ^13^C-NMR. The analysis, conducted by MS in a positive ion mode, showed a molecular ion peak of [M + H]^+^ of *m/z* 323.06876, with the theoretical *m/z* 323.069616 and mass error of −2.65 ppm (Figure 2A). As shown in Figure 2B, under negative ion mode, a molecular ion peak of [M − H]^−^ of *m/z* 321.05533 was detected with the theoretical *m/z* 321.055064 and mass error of 0.83 ppm. Moreover, as depicted in Figures S1 and S2, the position and number of chemical shifts in ^1^H-NMR and ^13^C-NMR data indicated that hapten SA10-X was successfully synthesized. 

*4-((4-Aminophenyl)sulfonamido)-2-methoxybenzoic acid* (**SA10-X**) ^1^H-NMR (300 MHz, DMSO-*d_6_*), *δ* (ppm) 12.67 (br, s, 1H, COOH), 9.66 (s, 1H, NH), 7.36-7.32 (dd, 2H, *J* = 12 Hz, CHar), 7.31 (s, 1H, CHar), 7.20-7.17 (d, 1H, *J* = 9 Hz, CHar), 7.01-6.98 (d, 1H, *J* = 9 Hz, CHar), 6.55-6.51 (dd, 2H, *J* = 12 Hz, CHar), 5.97 (br, s, 2H, NH_2_), 3.73 (s, 3H, CH_3_); ^13^C-NMR (300 MHz, DMSO-*d_6_*), *δ* (ppm) 166.84, 155.02, 152.90, 130.82, 128.78 (2X), 125.88, 124.37, 123.75, 121.39, 113.29, 112.71 (2X), 56.04. 

**Figure 3:**
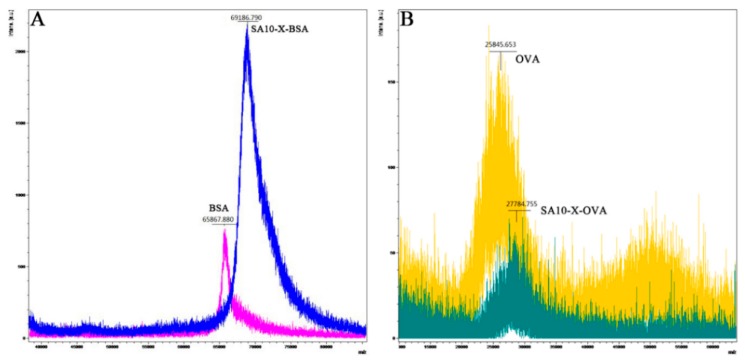
MALDI-TOF-MS result of conjugate SA10-X-BSA (**A**) and SA10-X-OVA (**B**).

The conjugation ratios of immunogen (SA10-X-BSA) and coating antigen (SA10-X-OVA) were estimated by measuring the hapten/carrier protein molar ratios, through MALDI-TOF-MS. Figure 3 show bioconjugates with molecular ion peaks that shifted behind their carrier proteins, indicating successful conjugation of hapten SA10-X to proteins. The coupling ratios were calculated as 10.3 and 6.02 for BSA and OVA conjugates, respectively. An appropriate coupling ratio is helpful to improve the affinity and specificity of the antibody, and the optimal coupling ratio is accepted to be 10–20 [23]. The immunogen SA10-X-BSA with a conjugation ratio of 10.3 was suitable for pAb production. 

### 2.3. Production and Specificity of pAb 

Three New Zealand white rabbits were immunized with SA10-X-BSA in this study. Rabbit #2 had the highest antiserum titer of 10,240,000 and the best sensitivity; it was sacrificed for further pAb purification. 

The optimal coating antigen was selected before determining the IC_50_ values and specificity of pAb against SAs. Many immunoassays can be optimized to achieve higher sensitivity based on the application of a heterologous antigen. Generally, structure-related haptens, haptens with different spacer arms, different carrier proteins, and different junction sites between the hapten and carrier protein can be used to prepare heterologous antigens. In heterologous systems, the binding affinity between the antibody and the competing antigen is lower than that of the target—a lower concentration of the analyte can compete with the coating antigen or enzyme-labeled antigen at a fixed concentration. This competition can improve detection limits. Here, we used 8 heterologous antigens to identify the optimal coating antigen (Table S1 in the Supporting Information). The results showed that binding between pAb and SA10-X-OVA was too strong. There was no obvious inhibition with 500 µg L^−1^ of SMZ. The BS-OVA with the lowest IC_50_ value was used as the best coating antigen. 

**Figure 4:**
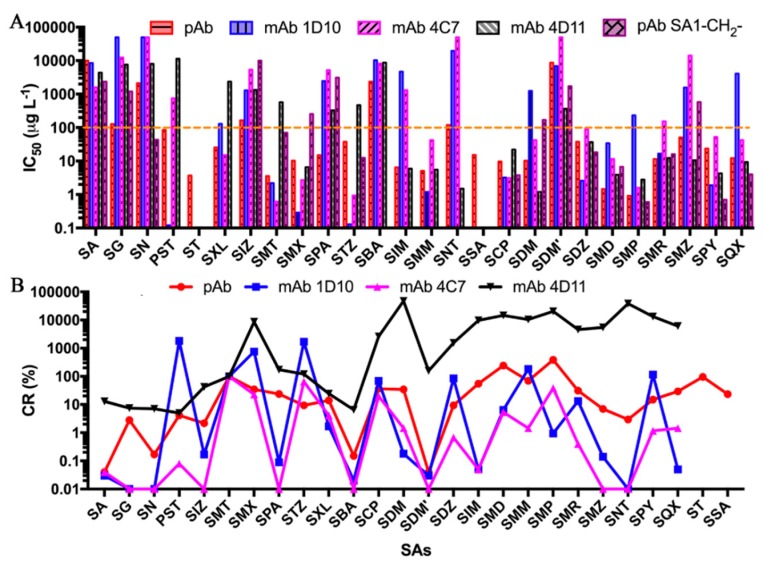
The IC_50_ (**A**) and corresponding cross-reactivity (CR). values (**B**) comparison between the pAb produced in this study and mAb 1D10, 4C7, 4D11 and pAb SA1-CH_2_- against SAs. PST, ST, SXL, SBA, SIM, SMM, SNT and SSA have not been detected in pAb SA1-CH_2_- based assay. And ST and SSA have not been detected in mAb 4C7 and 4D11 based assays. Hapten SA10-X, SMX, STZ, SA10 and SA1-CH_2_- were used for the preparation of pAb (this study), mAb 1D10, 4C7, 4D11 and pAb SA1-CH_2_-, respectively. The BS-OVA, SDZ-OVA, TS-OVA, CS-OVA and SA1-(CH_2_)_5_-HRP were used as the coating/competitive antigens for pAb (this study), mAb 1D10, 4C7, 4D11 and pAb SA1-CH_2_-, respectively.

Table S2 in the Supporting Information details IC_50_ values of SAs and their corresponding chemical structures. The results show that the IC_50_ values of 19 SAs were lower than 100 µg L^−1^, and demonstrate the superior performance of pAb elicited from the hapten SA10-X, with broad specificity and highly uniform affinity. Among these 19 SAs, SMT, ST, SMX, SPA, SXL, STZ, and PST are two-ring SAs containing a five-atom ring. These are recognized as well as SAs containing a six-atom ring. But for single-ring SAs, the affinity of pAb towards SA, SG, and SN was much lower than two-ring SAs similar to other antibodies prepared previously (Figure 4). Moreover, this antibody had the best recognition ability against SAs with methoxy group such as SMP, SMD, SMM, and SDM with IC_50_ values of 0.92–10.34 µg L^−1^ and SAs with methyl group such as SMT, ST, SIM, SMX, SMR, SXL, and SMZ (IC_50_ of 3.56–50.78 µg L^−1^). SCP had a chloro group substituted on the pyridazine ring and also showed high affinity (IC_50_ of 9.75 µg L^−1^) towards pAb. Even for SAs with a bulkier ring, such as SPA and SQX, had IC_50_ values of 15.01 and 12.18 µg L^−1^, respectively. These results concurred with our previous hypothesis on the design of hapten SA10-X to generate an antibody with broad specificity and highly uniform affinity, and indicated that high similarity between hapten and SAs in stereochemical and electronic aspects, an antibody with low IC_50_ values against these SAs will be obtained. However, SDM’ has a methoxy group at the *ortho-* position of the six-membered ring and had the worst binding affinity towards pAb. 

The analysis between pAb and previously produced mAb 1D10, 4C7 and 4D11 in IC_50_ and CR are shown in Figure 4 [17]. The mAb 1D10 and 4C7 showed higher affinity towards SAs containing a five-atom ring such as SMX and STZ than SAs containing a six-atom ring (Figure 4A). This is because hapten SMX and TS used to elicit mAb 1D10 and 4C7, respectively, are both two-ring haptens containing a five-atom ring. While, for mAb 4D11, the recognition profile towards SAs, containing a six-atom ring was very similar to the pAb. However, there was poor affinity of mAb 4D11 against SAs, containing a five-atom ring. The significant fluctuation of CR plots for mAb 1D10 and 4C7 showed that their affinities with SAs were variable (Figure 4B). The mAb 4D11 had a steady curve towards SAs containing a six-atom ring, but dropped rapidly towards SAs containing a five-atom ring. Only pAb elicited from hapten SA10-X with CR in the range of 2.16–386.96% (except SA, SN, SBA, and SDM’) had a smooth curve. This demonstrates its more uniform affinity relative to mAbs 1D10, 4C7, and 4D11. 

The produced antibodies and their recognition profiles have been summarized in Table S2 in the Supporting Information. For pAb HS, SAB, and SA1-CH_2_ raised against single-ring haptens, the performance has been described above, which indicated the single-ring haptens are not suitable for the generic antibody production [14,20,24]. Regarding mAb 27G3, pAb SA2’ and D3 raised against two-ring SAs, their recognition abilities have been mentioned in the above content [11,14,22]. The Sulfa-1 was the first produced generic mAb against SAs, but its overall recognition ability towards SAs is weak with the IC_50_ values ranged from 1.41–200,000 µg L^−1^ [9]. The mAb 4E10 showed better affinity towards two-ring SAs containing a five-atom ring with the IC_50_ values ranged from 2–130 µg L^−1^ than two-ring SAs containing a six-atom ring [11]. So as the mAb 3B5B10E3, which depicted the IC_50_ values of 600 µg L^−1^ for SMR and 1050 µg L^−1^ for SMZ both containing a six-atom ring [12]. Most of the IC_50_ values of mAb 4E5, rAb and mAb produced by Chen et al. were below 100 µg L^−1^, which revealed the high sensitivity of these antibodies [13,15,16]. But the uniform CR of mAb 4E5, rAb, and mAb produced by Chen et al. is inferior when compared with the pAb in this study. Therefore, in this study, a superior pAb with broad specificity and highly uniform affinity was produced. 

### 2.4. CoMFA for pAb 

**Figure 5:**
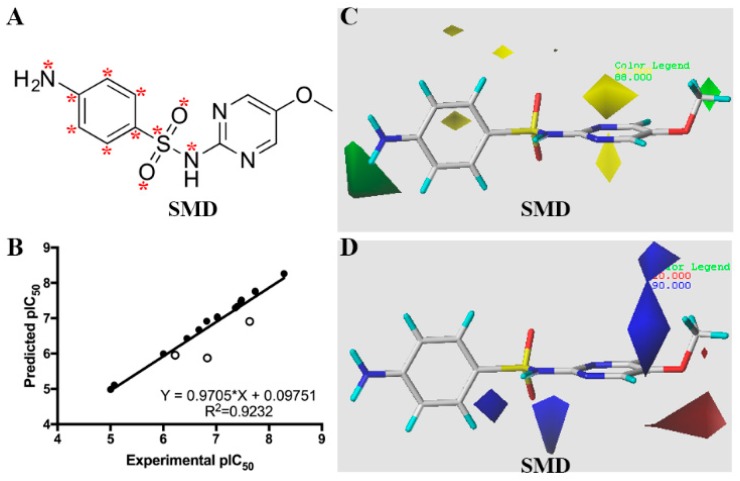
CoMFA results for pAb. (**A**) Template molecule sulfameter (SMD). Red asterisk shows the common backbone used for alignment. (**B**) Plot of predicted pIC_50_ values versus measured pIC_50_. The solid circles indicate SAs in the training set, and hollow circles depict molecules in the test set. The contour plots of the CoMFA steric fields (**C**) and electrostatic fields (**D**). Green and yellow contours indicate bulky groups with favorable and unfavorable regions, respectively. Red and blue contours represent negative and positive charged groups favorable regions, respectively.

The CR analysis above was only based on two-dimensional structures of SAs. As mentioned during the design of SA10-X, the stereochemical and electronic properties of SAs serve important roles during interaction with antibodies. To deepen our understanding on the specificity of produced pAb and find clues for novel hapten design, we next extended this work to a three-dimensional CoMFA model for pAb. It had a *R*_cv_^2^ of 0.651 with four principal components. As shown in Figure 5A, SMD (pIC_50_, 8.281) was used as the template molecule. A non-cross-validated *R*^2^ value was 0.999, and SEE was 0.048, and F was 1376.09 for the training set. This illustrated its robust and high predictive ability of developed model (Figure 5B). The contributions of the steric, and electrostatic fields were 38.2%, and 61.8%, respectively. This result indicated that the electrostatic profiles of the SA groups play an important role during pAb recognition. 

As revealed in Figure 5C, green contour was showed up near the methoxypyrimidine group of SMD. The affinity of SDZ (pIC_50_, 6.820) was lower than SMD towards pAb due to the lack of a bulky methoxy group. The activity of SMD was higher than SDM (pIC_50_, 7.477), which is mainly because yellow contour was around the bulky methoxy group on the pyrimidine ring of SDM. Moreover, since negatively charged favorable red contour was located near the methoxy group of SMD, this contributed to its affinity higher than SDZ (Figure 5D). Blue contours were distributed near the methoxypyrimidine group of SMD, which indicate lower activity if negative charged atoms were substituted on the pyrimidine ring. For example, SMM has a nitrogen instead of a carbon atom and a pIC_50_ of 7.738; SDM also has a lower pIC_50_ value. The results revealed that due to the differences in spatial and electron density of the heterocyclic ring between SAs, the pAb had variable IC_50_ values against different SAs. Based on CoMFA model, the future design of novel haptens will introduce one or two nitrogen atoms on the second benzene ring along with methyl or methoxy groups. This will likely produce antibodies with ultra-uniform affinity for SAs [2]. 

### 2.5. Optimization of ELISA 

Many factors can influence the performance of an immunoassay [25]. We evaluated 9 parameters to fully improve the performance of multianalyte ELISA for multi-SAs residue detection (from coating plates to color development; Table S3 in the Supporting Information). The maximum optical density value (B_0_), IC_50_ value, and B_0_/IC_50_ of SMZ, were utilized to investigate the effects of these factors. The optimal coating condition used carbonate buffer (pH 9.6) as the coating buffer, and we incubated the plate at 4 °C overnight, because this gave the lowest IC_50_. The best blocking buffer was PBS with 5% FBS; the plate was held at 37 °C with 2 h for blocking. In the competitive assay, a dilution buffer with 0.01 M PB containing 2.6 mM of KCl and 50 mM of NaCl at pH 7.4 and no Tween 20 gave the lowest IC_50_. Incubation times of 30 min for competition, and 30 min for incubation with HRP-labeled secondary antibody, were used in the following work. 

### 2.6. Detection of Milk Samples 

**Figure 6:**
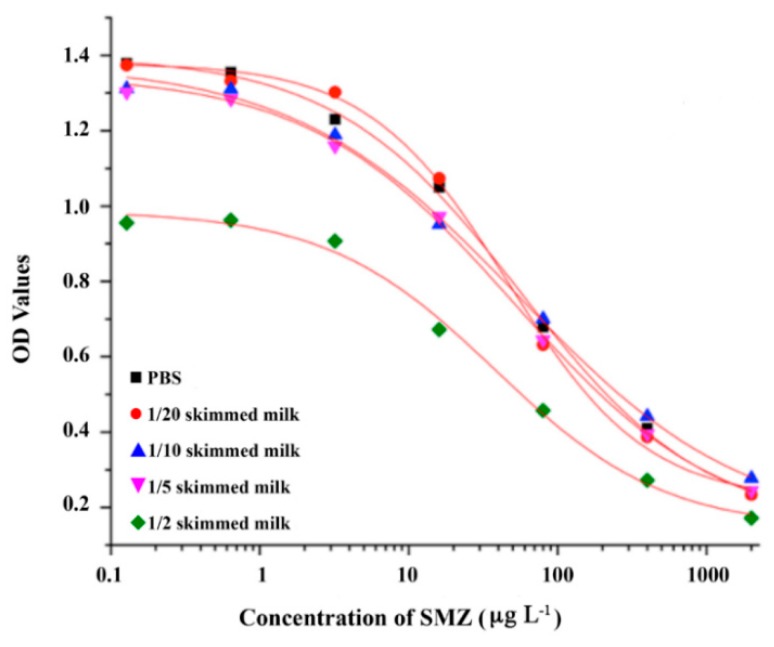
Matrix effects of phosphate-buffered saline (PBS) diluted skimmed milk using sulfamethazine (SMZ) as the reference molecule. Vertical bars denote the SE for triplicate measurements.

Rapid screening implies minimal sample pre-treatment, and thus direct dilution was used for milk samples. The SMZ standard curves were developed in buffer and compared with the curves prepared in milk samples diluted 2, 5, 10, and 20 times. As shown in Figure 6, when the samples were diluted 2–20 times, the IC_50_ values for SMZ were 38.11, 50.84, 54.98, and 55.24 µg L^−1^. The matrix effect was removed by 5-, 10- and 20-fold dilution, and thus 5-fold dilution was selected for further work. The limit of detection (LOD) was determined as the mean of the blank samples (n=20) plus 3SD [26]. The LODs for SMM, SMX, SQX, SDM, and SMZ were 1.00, 1.25, 2.95, 3.35, and 6.10 µg L^−1^, respectively, in skimmed milk samples. 

This developed ELISA immunoassay was then used to quantify SDM, SMM, SMX, SMZ, and SQX in spiked skimmed milk samples with concentrations near the MRLs. The recoveries and coefficients of variation (CV) are listed in Table 1. The average recoveries for these 5 SAs were 72.0–107.5%, with CVs ranging from 1.0–14.1%. Therefore, the ELISA method developed here, based on our prepared pAb, meets the requirements for a screening detection method and can be used for class-specific multi-SAs residue measurement in milk. 

## 3. Materials and Methods 

### 3.1. Reagents, Materials and Apparatus 

The *N*-acetylsulfanilyl chloride, *N,N*-dimethylformamide (DMF), methyl 4-amino-2-methoxybenzoate, triethylamine, 4-(dimethylamino)pyridine (DMAP), tetrahydrofuran (THF), *N,N’*-dicyclohexylcarbodiimide (DCC), *N*-hydroxysuccinimide (NHS), ovalbumin (OVA), bovine serum albumin (BSA), Freund’s complete adjuvant (FCA) and Freund’s incomplete adjuvant (FIA) were purchased from MilliporeSigma (St. Louis, MO, USA). Fetal bovine serum (FBS) was obtained from Thermo Fisher Scientific (Waltham, MA, USA). Standards phthalylsulfathiazole (PST), sulfabenzamide (SBA), sulfacetamide (SA), sulfachloropyridazine (SCP), sulfadiazine (SDZ), sulfadimethoxine (SDM), sulfadoxine (SDM’), sulfaguanidine (SG), sulfamerazine (SMR), sulfameter (SMD), sulfamethazine (SMZ), sulfamethizole (SMT), SMX, sulfamethoxypyridazine (SMP), sulfamethylthiazole (ST), sulfamonomethoxine (SMM), sulfamoxole (SXL), sulfanilamide (SN), sulfanitran (SNT), sulfaphenazole (SPA), sulfapyridine (SPY), sulfaquinoxaline (SQX), sulfasalazine (SSA), sulfathiazole (STZ), sulfisomidine (SIM) and sulfisoxazole (SIZ) were acquired from MilliporeSigma (St. Louis, MO, USA) or LGC Standards (Teddington, Middlesex, UK). All other chemicals and solvents were of analytical grade or better, and were obtained from Sinopharm Chemical Reagent Co., Ltd. (Beijing, China). Transparent 96-well microtiter ELISA plates were purchased from Corning (Corning, NY, USA). The microplate reader was obtained from PerkinElmer (Waltham, MA, USA). Solutions and buffers were prepared with water purified using a Milli-Q system from MilliporeSigma (St. Louis, MO, USA). 

### 3.2. Buffers and Solutions 

The following buffers were used: (1) Coating buffer (CB) was 0.05 M carbonate buffer, pH 9.6; (2) blocking buffer consisted of 0.01 M phosphate-buffered saline (PBS) and 5% FBS, pH 7.4; (3) washing buffer was 0.01 M PBS containing 0.1% Tween 20; (4) standard dilution and antibody dilution buffer was 0.01 M PB containing 2.6 mM of KCl and 50 mM of NaCl, pH 7.4; (5) 2 M H_2_SO_4_ was used as the stopping reagent in the ELISA protocol. 

Stock solutions of each SAs (1 g L^−1^) were prepared by dissolving an appropriate amount of each standard in DMF with storage at −20 °C. Working standards (0.01–500,000 µg L^−1^) for each SA were prepared by diluting the stock solution in dilution buffer. 

### 3.3. Hapten SA10-X and Bioconjugates Synthesis 

Hapten SA10-X was synthesized according to a previous report with some modification [9]. As shown in Figure 1, the first step is dissolving 1 mmol methyl 4-amino-2-methoxybenzoate into 4 mL THF, followed by the addition of 150 μL triethylamine and 0.5 mmol DMAP. After mixing, 1 mmol *N*-acetylsulfanilyl chloride was added, and the mixture kept reacting at 60 °C for 2–3 h. Following the completion of the reaction, the solution was poured into 200 mL deionized water and extracted with dichloromethane 2–3 times. The organic phase was dried over Na_2_SO_4_, and concentrated in vacuum. The second step dissolved 1.2 g NaOH into 50 mL deionized water. This was then transferred to a flask containing the concentrated reactant above. After reacting at 95 °C for 2–3 h, the solution was adjusted to pH 4.0 and extracted with ethyl acetate 2–3 times. The organic phase was dried over Na_2_SO_4_, and concentrated in vacuum. In the final step, the 4-[(4-aminophenyl) sulfonylamino]-2-methoxybenzoic acid product was obtained via purification with silica-gel chromatography. The purified hapten was obtained in 24.8% yield and named SA10-X; this was confirmed with UHPLC-MS/MS, ^1^H-NMR and ^13^C-NMR. 

The hapten SA10-X contains a carboxyl group, that was used for covalent conjugated to BSA and OVA via the NHS ester method, according to our previous study [17]. These were used as the immunogen (SA10-X-BSA), and coating antigen (SA10-X-OVA), respectively. Briefly, hapten SA10-X (40 μmol), DCC (60 μmol), and NHS (60 μmol) were dissolved in 0.5 mL DMF, and the mixture was stirred at room temperature for 1 h. After centrifugation, the supernatant was added dropwise under mixing to 6 mL of 1 μmol BSA or OVA in CB (pH 9.4). The mixture was stirred overnight at 4 °C, dialyzed against PBS (pH 7.4) for 72 h, and then stored as aliquots at −20 °C until its use. The conjugations were confirmed with MALDI-TOF-MS, and the coupling ratio was calculated. 

### 3.4. Polyclonal Antibody Generation 

New Zealand white rabbits were immunized sub-cutaneously with 1 mg of immunogen in FCA. One mg of SA10-X-BSA in FIA was later used to boost immunization at 3-weeks intervals, 5 times. Serum was collected from the ear vein 7 days after the third immunization; this was later tested by ELISA as detailed below. The rabbit with the lowest IC_50_ values and uniform CR of antiserum against SAs was sacrificed for the preparation of pAb. Ammonium sulfate precipitation was used to isolate pAb from rabbit sera. All animal work complied with the guidelines for the care and use of laboratory animals as detailed by the Chinese laws and guidelines (GKFCZ2001545) and Animal Care Center of China Agricultural University (2012-SYXK-0037). 

### 3.5. Molecular Modeling Study 

For a better understanding of the CR of pAb against SAs, a comparative molecular field analysis (CoMFA) was used in this study to figure out the relationship between SAs structure and the corresponding affinity (pIC_50_) towards pAb. The 3D conformations of the SAs were retrieved from the PubChem Database. According to our previous study, one SA with the highest binding affinity was selected as the template [27]. Three SAs were selected as the test set, and the others assembled the training set. Variables representing the steric and electrostatic fields of each SA were calculated via the Sybyl program, followed by the CoMFA model developed by principal components analysis with the leave-one-out method. 

### 3.6. Competitive Indirect ELISA Procedure 

Polystyrene 96-well microtiter plates were coated with coating antigen in CB (100 μL well^−1^) and incubated at 4 °C overnight. The plates were washed three times with washing buffer and then blocked with blocking buffer (150 μL well^−1^) at 37 °C for 2 h. Then, antiserum (50 μL well^−1^) and analytes (or buffer; 50 μL well^−1^) were added successively and incubated at 37 °C for 30 min. The plates were washed 3X with washing buffer, followed by goat anti-rabbit IgG-HRP (1:5,000 in PBS, 100 μL well^−1^) pipetted and incubated at 37 °C for 30 min. After the plates were washed 3X, a substrate solution (100 μL well^−1^) was added and incubated at 37 °C for 10 min followed by the addition of a stopping solution (50 μL well^−1^). The absorbance of each microwell was measured at 450 nm with a plate reader. A four-parameter logistic function was used to fit these data into sigmoidal curves. The CR values calculated referred to the IC_50_ value of SMT. 

### 3.7. Assay Optimization 

Coating antigens BS-OVA, HS-OVA, SA10-OVA, SG-OVA, SMZ-OVA, ST-OVA, and TS-OVA, prepared previously in our laboratory, were used for screening the optimal coating antigen for pAb in this study [17]. The coating conditions, blocking buffers, blocking time, components of dilution buffer (pH values, NaCl, Tween 20), competitive time, and reaction time of IgG-HRP used here were optimized with SMZ as a reference analyte. 

### 3.8. Milk Samples Analysis 

SAs-free skimmed milk samples were acquired from the National Reference Laboratory for Veterinary Drug Residues (Beijing, China). SMZ was selected as the reference molecule to assess the matrix effects. Non-specific interferences, generated by the skimmed milk, were evaluated by comparing the SMZ standard curves in skim milk at several dilutions with the curves in PBS. All measurements were completed in triplicate. The SDM, SMM, SMX, SMZ, and SQX were spiked into the milk at 25, 100, and 400 μg L^−1^, respectively. The accuracy and precision of this assay were determined via these recovery data. 

## 4. Conclusions

In this study, a novel hapten SA10-X was rationally designed and the produced pAb has affinity for 19 SAs below 100 μg L^−1^, with superior group-specific and highly uniform performance. The results confirmed our hypothesis that the hapten should have two rings to elicit a strong antibody response against two-ring SAs. The introduction of a methoxy group on the heterocyclic ring could lead to anti-SAs antibodies with more sensitive and uniform affinity. Specificity comparisons between different antibodies and CoMFA results provided us more clues for future hapten design and ultra-uniform antibody production. This pAb was used for a class-specific ELISA for the detection of SAs in skimmed milk after five-fold dilution. After optimization, the LODs for SMM, SMX, SQX, SDM, and SMZ were 1.00, 1.25, 2.95, 3.35, and 6.10 µg L^−1^, respectively. The average recoveries were 72.0–107.5% with CVs lower than 14.1%. This tool has broad specificity, highly uniform affinity, good sensitivity, and low cost, making it ideal for multianalyte analysis. 

## Figures and Tables

**Table 1:** Recoveries of milk samples spiked with Sulfonamides (SAs) (*n* = 3).

SAs	Spiked Concentration (µg L^−1^)	Batch #1		Batch #2		Batch #3		CV (%)
		Recovery (%)	CV (%)	Recovery (%)	CV (%)	Recovery (%)	CV (%)	
SDM	25	80.2	8.6	81.9	6.6	81.0	4.8	1.0
	100	102.0	2.6	104.9	5.3	98.5	4.1	3.1
	400	91.3	11.5	93.6	6.4	100.3	6.5	4.9
SMM	25	79.9	1.5	85.3	5.4	86.8	4.7	4.3
	100	96.7	10.7	100.8	5.8	97.5	6.7	2.2
	400	100.9	8.4	99.5	10.1	93.3	10.1	4.1
SMX	25	98.9	8.9	100.2	12.8	98.0	7.4	1.1
	100	103.3	7.6	107.5	11.9	100.1	3.8	3.6
	400	72.0	3.5	74.7	2.5	80.7	9.3	5.9
SMZ	25	90.2	5.8	91.7	8.8	95.1	13.6	2.7
	100	97.0	4.4	93.7	3.5	88.8	6.1	4.4
	400	97.8	14.1	98.6	11.4	102.7	7.8	2.6
SQX	25	94.8	2.6	99.2	1.1	95.3	3.5	2.5
	100	94.7	3.1	91.3	3.4	100.7	8.0	5.0
	400	101.3	2.5	97.3	3.5	96.0	3.4	2.8

## Supplementary Materials

The supplementary materials are available online.

## References

[1] Cháfer-Pericás C., Maquieira Á., Puchades R., Miralles J., Moreno A. (2011). Multiresidue determination of antibiotics in feed and fish samples for food safety evaluation. Comparison of immunoassay vs LC-MS-MS. Food Control.

[2] Chen Y., Guo L., Liu L., Song S., Kuang H., Xu C. (2017). Ultrasensitive immunochromatographic strip for fast screening of 27 sulfonamides in honey and pork liver samples based on a monoclonal antibody. J. Agric. Food Chem..

[3] Jiang W., Wang Z., Beier R.C., Jiang H., Wu Y., Shen J. (2013). Simultaneous determination of 13 fluoroquinolone and 22 sulfonamide residues in milk by a dual-colorimetric enzyme-linked immunosorbent assay. Anal. Chem..

[4] PRC Ministry of Agriculture (2002). Bulletin No 235, Maximum Residue Limits of Veterinary Medicinal Products in Foodstuffs of Animal Origin.

[5] Food and Drug Administration (2018). Tolerances for Residues of New Animal Drugs in Food.

[6] European Union (2010). Commission regulation (EU) No 37/2010 of 22 December 2009 on pharmacologically active substances and their classification regarding maximum residue limits in foodstuffs of animal origin. Off. J. Eur. Union.

[7] Cháfer-Pericás C., Maquieira Á., Puchades R. (2010). Fast screening methods to detect antibiotic residues in food samples. Trends Anal. Chem..

[8] Li Y., Sun Y., Beier R.C., Lei H., Gee S., Hammock B.D., Wang H., Wang Z., Sun X., Shen Y., Yang J., Xu Z. (2017). Immunochemical techniques for multianalyte analysis of chemical residues in food and the environment: A review. Trends Anal. Chem..

[9] Muldoon M.T., Font I.A., Beier R.C., Holtzapple C.K., Young C.R., Stanker L.H. (1999). Development of a cross-reactive monoclonal antibody to sulfonamide antibiotics: Evidence for structural conformation-selective hapten recognition. Food Agric. Immunol..

[10] Haasnoot W., Cazemier G., Pre J.D., Kemmers-Voncken A., Bienenmann-Ploum M., Verheijen R. (2000). Sulphonamide antibodies: From specific polyclonals to generic monoclonals. Food Agric. Immunol..

[11] Haasnoot W., Pre J.D., Cazemier G., Kemmers-Voncken A., Verheijen R., Jansen B.J.M. (2000). Monoclonal antibodies against a sulfathiazole derivative for the immunochemical detection of sulfonamides. Food Agric. Immunol..

[12] Cliquet P., Cox E., Haasnoot W., Schacht E., Goddeeris B.M. (2003). Generation of group-specific antibodies against sulfonamides. J. Agric. Food Chem..

[13] Zhou Q., Peng D., Wang Y., Pan Y., Wan D., Zhang X., Yuan Z. (2014). A novel hapten and monoclonal-based enzyme-linked immunosorbent assay for sulfonamides in edible animal tissues. Food Chem..

[14] Franek M., Diblikova I., Cernoch I., Vass M., Hruska K. (2006). Broad-specificity immunoassays for sulfonamide detection: Immunochemical strategy for generic antibodies and competitors. Anal. Chem..

[15] Korpimäki T., Hagren V., Brockmann E.-C., Tuomola M. (2004). Generic lanthanide fluoroimmunoassay for the simultaneous screening of 18 sulfonamides using an engineered antibody. Anal. Chem..

[16] Chen Y., Liu L., Xu L., Song S., Kuang H., Cui G., Xu C. (2017). Gold immunochromatographic sensor for the rapid detection of twenty-six sulfonamides in foods. Nano Res..

[17] Wang Z., Beier R.C., Sheng Y., Zhang S., Jiang W., Wang Z., Wang J., Shen J. (2013). Monoclonal antibodies with group specificity toward sulfonamides: Selection of hapten and antibody selectivity. Anal. Bioanal. Chem..

[18] Wang Z., Liang X., Wen K., Zhang S., Li C., Shen J. (2015). A highly sensitive and class-specific fluorescence polarisation assay for sulphonamides based on dihydropteroate synthase. Biosens. Bioelectron..

[19] Li J., Li X., Yuan J., Wang X. (2000). Determination of sulfonamides in swine meat by immunoaffinity chromatography. J. AOAC Int..

[20] Ermolenko D.N., Eremin S.A., Mart’ianov A.A., Zherdev A.V., Dzantiev B.B. (2007). A new generic enzyme immunoassay for sulfonamides. Anal. Lett..

[21] Adrian J., Font H., Diserens J.-M., Sánchez-Baeza F., Marco M.-P. (2009). Generation of broad specificity antibodies for sulfonamide antibiotics and development of an enzyme-linked immunosorbent assay (ELISA) for the analysis of milk samples. J. Agric. Food Chem..

[22] Zhang H., Duan Z., Wang L., Zhang Y., Wang S. (2006). Hapten synthesis and development of polyclonal antibody-based multi-sulfonamide immunoassays. J. Agric. Food Chem..

[23] Smith D.S., Hassan M., Nargessi R.D., Wehry E.L. (1981). Principles and practice of fluoroimmunoassay procedures. Modern Fluorescence Spectroscopy.

[24] Wang Z., Li Y., Liang X., Zhang S., Shi W., Shen J. (2013). Forcing immunoassay for sulfonamides to higher sensitivity and broader detection spectrum by site heterologous hapten inducing affinity improvement. Anal. Methods.

[25] Yu X., Tao X., Shen J., Zhang S., Cao X., Chen M., Wang W., Wang Z., Wen K. (2015). A one-step chemiluminescence immunoassay for 20 fluoroquinolone residues in fish and shrimp based on a single chain Fv–alkaline phosphatase fusion protein. Anal. Methods.

[26] Yu X., Wen K., Wang Z., Zhang X., Li C., Zhang S., Shen J. (2016). General bioluminescence resonance energy transfer homogeneous immunoassay for small molecules based on quantum dots. Anal. Chem..

[27] Wang Z., Zhu Y., Ding S., He F., Beier R.C., Li J., Jiang H., Feng C., Wan Y., Zhang S. (2007). Development of a monoclonal antibody-based broad-specificity ELISA for fluoroquinolone antibiotics in foods and molecular modeling studies of cross-reactive compounds. Anal. Chem..

