# Supplementary material for: A Class-Selective Immunoassay for Sulfonamides Residue Detection in Milk Using a Superior Polyclonal Antibody with Broad Specificity and Highly Uniform Affinity
Source: Molecules. 2019 Jan 26;24(3):443. doi: 10.3390/molecules24030443 (PMC6384663; doi:10.3390/molecules24030443)
Supplement: Supplementary file 1 [file molecules-24-00443-s001.pdf]

A Class-Selective Immunoassay for Sulfonamides Residue Detection in Milk using a Superior Polyclonal Antibody with Broad Specificity and Highly Uniform Affinity

Chenglong Li [1], Xiangshu Luo [1], Yonghan Li [2], Huijuan Yang [1], Xiao Liang [3], Kai Wen [1], Yanxin Cao [1], Chao Li [1], Weiyu Wang [1], Weimin Shi [1], Suxia Zhang [1], Xuezhi Yu [1,*] and Zhanhui Wang [1]

[1] Beijing Advanced Innovation Center for Food Nutrition and Human Health, College of Veterinary Medicine, China Agricultural University, Beijing Key Laboratory of Detection Technology for Animal-Derived Food Safety, Beijing Laboratory of Food Quality and Safety, 100193 Beijing, People's Republic of China
[2] Henan Animal Health Supervision Institute, 450008 Zhengzhou, People's Republic of China
[3] College of Veterinary Medicine, Qingdao Agricultural University, 266109 Qingdao, People's Republic of China
* Correspondence: yu51422396@126.com; Tel.: +86-10-6273 4565; Fax: +86-10-6273 1032

Figure Captions

Figure S1. ^{1}H-NMR result of hapten SA10-X

Figure S2. ^{13}C-NMR result of hapten SA10-X

Table S1.

Coating antigens selecting result of pAb

Coating antigens	Coating antigens dilution	pAb dilution	B_0	B (100 μg L^{-1})	IC_{50} of SMZ (μg L^{-1})
BS-OVA	1:160,000	1:100,000	1.950	1.067	65.5
TS-OVA	1:5,000	1:20,000	1.749	1.188	174.3
ST-OVA	1:20,000	1:40,000	1.304	0.886	235.8
SA10-X-OVA	1:500,000	1:200,000	1.596	1.538	>500
SMZ-OVA	1:10,000	1:10,000	0.245	NA	NA
SG-OVA	1:10,000	1:10,000	0.235	NA	NA
HS-OVA	1:10,000	1:10,000	0.150	NA	NA
SA10-OVA	1:10,000	1:10,000	0.134	NA	NA

NA, not available

Table S2.

IC$_{50}$ values (μg L^{-1}) of SAs against pAb in this study and other antibodies in literatures

SAs	Structures	pAb	pAb HS [1]	pAb SAB [2]	pAb SA1-CH$_2$ [3]	27G3 [4]	pAb SA2' [3]	pAb D3 [5]	Sulfa-1 [6]	4E10 [4]	3B5B 10E3 [7]	4E5 [8]	rAb [9]	Chen [10]
**Single-ring SAs**														
SA	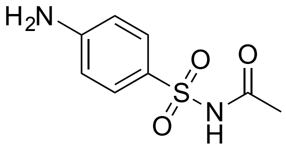	10,000	10,000	ND	2367	1800	452	ND	14,349	10,000	ND	100	7.9	41.16
SG	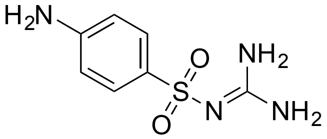	127.74	8614	63	1201	500	731	ND	ND	10,000	ND	100	12	30.24
SN	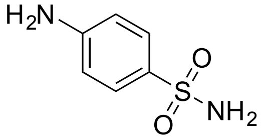	2122.4	7524	108	43.5	ND	19.0	ND	60,000	ND	ND	100	1800	90.18
**Two-ring SAs containing a five-atom ring**														
PST	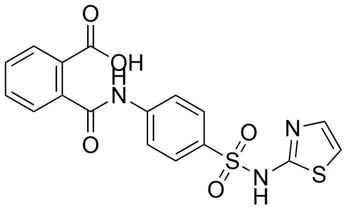	85.69	6531	ND	ND	ND	ND	ND	ND	ND	ND	ND	ND	60.16
SIZ	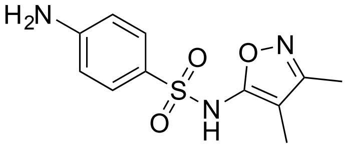	164.53	10,000	ND	10,000	250	95.6	200	200,000	80	350	51	0.51	35.62

SMT	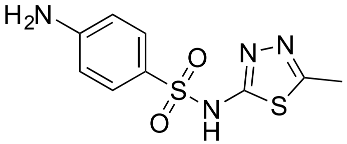	3.56	113.4	399	70.3	0.6	8.8	12.8	70,000	2	ND	39	0.019	0.08
SMX	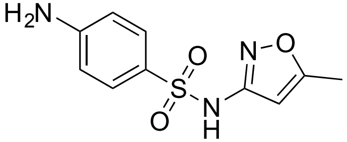	10.31	305.4	88	255	150	0.4	135	60,000	30	9	25	0.78	0.15
SPA	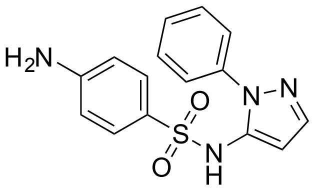	15.01	3024	ND	3098	ND	49.5	ND	ND	ND	ND	ND	0.39	28.27
ST	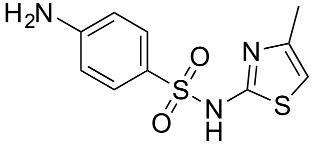	3.71	ND	ND	ND	ND	ND	ND	ND	ND	ND	ND	ND	ND
STZ	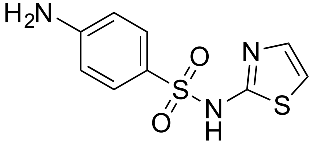	37.7	241.1	24	12.5	10	13.7	2	322	130	30	3.2	0.21	0.39
SXL	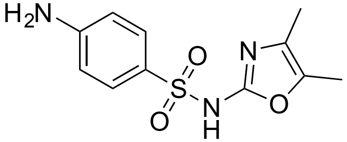	25.51	1590	ND	ND	ND	ND	150	ND	ND	ND	ND	ND	29.91
**Two-ring SAs containing a six-atom ring**														
SBA	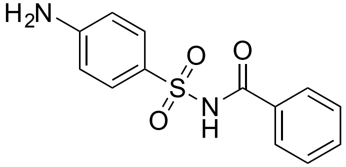	2349	111.8	ND	ND	ND	ND	ND	100,000	ND	ND	ND	ND	26.69

SCP	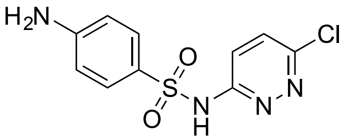	9.75	786.9	23	3.8	4	10.7	14	4360	4	20	7.3	0.072	0.39
SDM	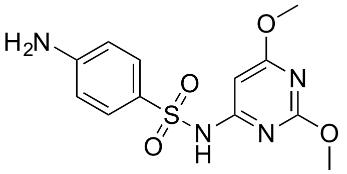	10.34	345.8	137	171	250	6.1	190	15,724	100	100	5.3	9.9	3.45
SDM'	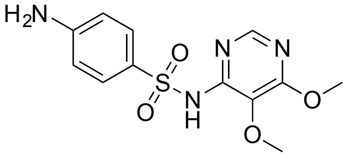	8786.2	5105	ND	1731	30	426	31	ND	800	ND	0.52	0.32	ND
SDZ	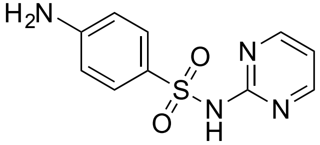	37.88	1282	36	18.2	80	19.5	32	9763	80	30	0.92	0.51	4.89
SIM	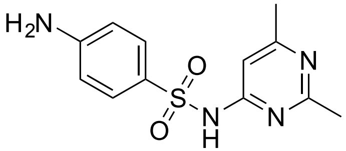	6.49	1302	ND	ND	ND	ND	1.3	3136	ND	ND	1.9	ND	48.39
SMD	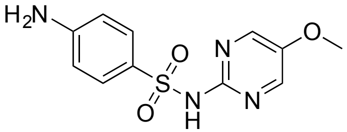	1.47	534.3	ND	6.7	ND	2.7	3.2	ND	ND	ND	0.76	ND	0.81
SMM	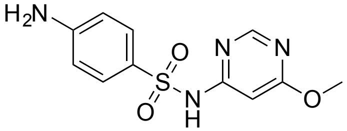	5.12	512.4	ND	ND	ND	ND	ND	ND	ND	ND	1.1	0.62	4.51
SMP	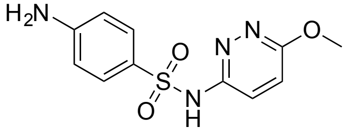	0.92	119.5	1.6	0.6	15	0.6	8.4	ND	13	ND	7.4	0.18	0.57

SMR	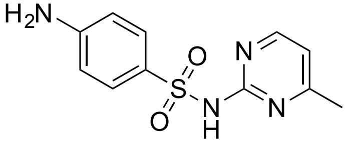	11.5	1419	18.8	16.1	500	36.2	19	3743	250	600	1.4	0.65	1.53
SMZ	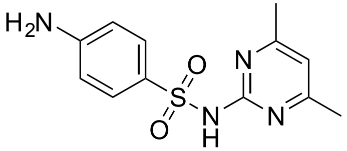	50.78	8337	118	582	8000	311	130	70,000	7000	1050	5.8	13	18.79
SNT	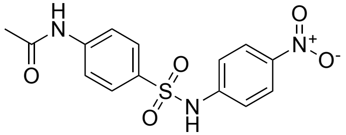	119.9	47.2	ND	ND	ND	ND	ND	1.41	ND	ND	ND	10	37.16
SPY	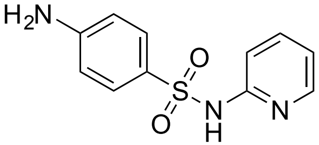	23.68	124.4	ND	0.7	30	0.4	10	22.8	150	350	100	0.26	5.09
SQX	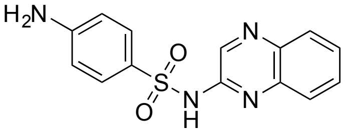	12.18	1053	455	4	1200	1.4	90	25,000	3000	ND	3.8	7.1	39.12
SSA	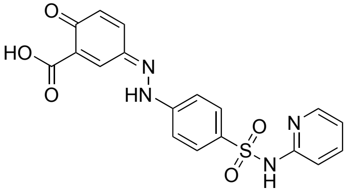	15.16	ND	ND	ND	ND	ND	ND	4305	ND	ND	ND	1100	80.05

ND, not detected; rAb, recombinant antibody

Table S3.

Optimized parameters of indirect competitive ELISA based on pAb using SMZ as reference analyte

Factors	Conditions	B_0	IC_{50} ($\mu g\ L^{-1}$)	B_0/IC_{50}	Factors	Conditions	B_0	IC_{50} ($\mu g\ L^{-1}$)	B_0/IC_{50}
Coating solutions	**CB**	**1.342**	**60.3**	**0.0223**	NaCl (M)	0	1.473	68.8	0.0214
	PBS	1.275	62.8	0.0203		**0.05**	**1.337**	**50.3**	**0.0266**
	BB	0.2	NA			0.15	1.246	54.5	0.0229
Coating condition	37°C 2 h	1.342	60.3	0.0223		0.3	1.087	57.2	0.0190
	**4°C overnight**	**1.357**	**55.5**	**0.0245**		0.5	0.875	61.7	0.0142
	37°C 2 h+4°C overnight	1.477	57.2	0.0258					
Blocking buffer	**5% FBS**	**1.349**	**51.8**	**0.0260**	Tween-20 (‰)	**0**	**1.373**	**51.8**	**0.0265**
	0.25% casein	1.009	52.6	0.0192		0.05	1.634	63.3	0.0258
	5% skimmed milk	0.896	54.9	0.0163		0.1	1.845	74.5	0.0248
						0.5	1.833	97.2	0.0189
						2	1.921	101.5	0.0189
Blocking time	30 min	1.527	78.9	0.0194	Competitive time	15min	0.816	62.5	0.0131
	1 h	1.435	57.3	0.0250		**30 min**	**1.357**	**50.4**	**0.0269**
	**2 h**	**1.314**	**53.2**	**0.0247**		45 min	1.364	53.2	0.0256
	4 h	1.266	53.5	0.0237		60 min	1.387	63.3	0.0219
pH	5.0	1.079	75.3	0.0143	Reaction time of IgG-HRP	15 min	0.735	57.2	0.0128
	6.0	1.363	63.5	0.0215		**30 min**	**1.349**	**50.8**	**0.0266**
	**7.4**	**1.374**	**54.2**	**0.0254**		45 min	1.577	53.7	0.0294
	8.0	1.382	67.7	0.0204		60 min	1.723	55.4	0.0311
	9.0	0.977	89.2	0.0110					

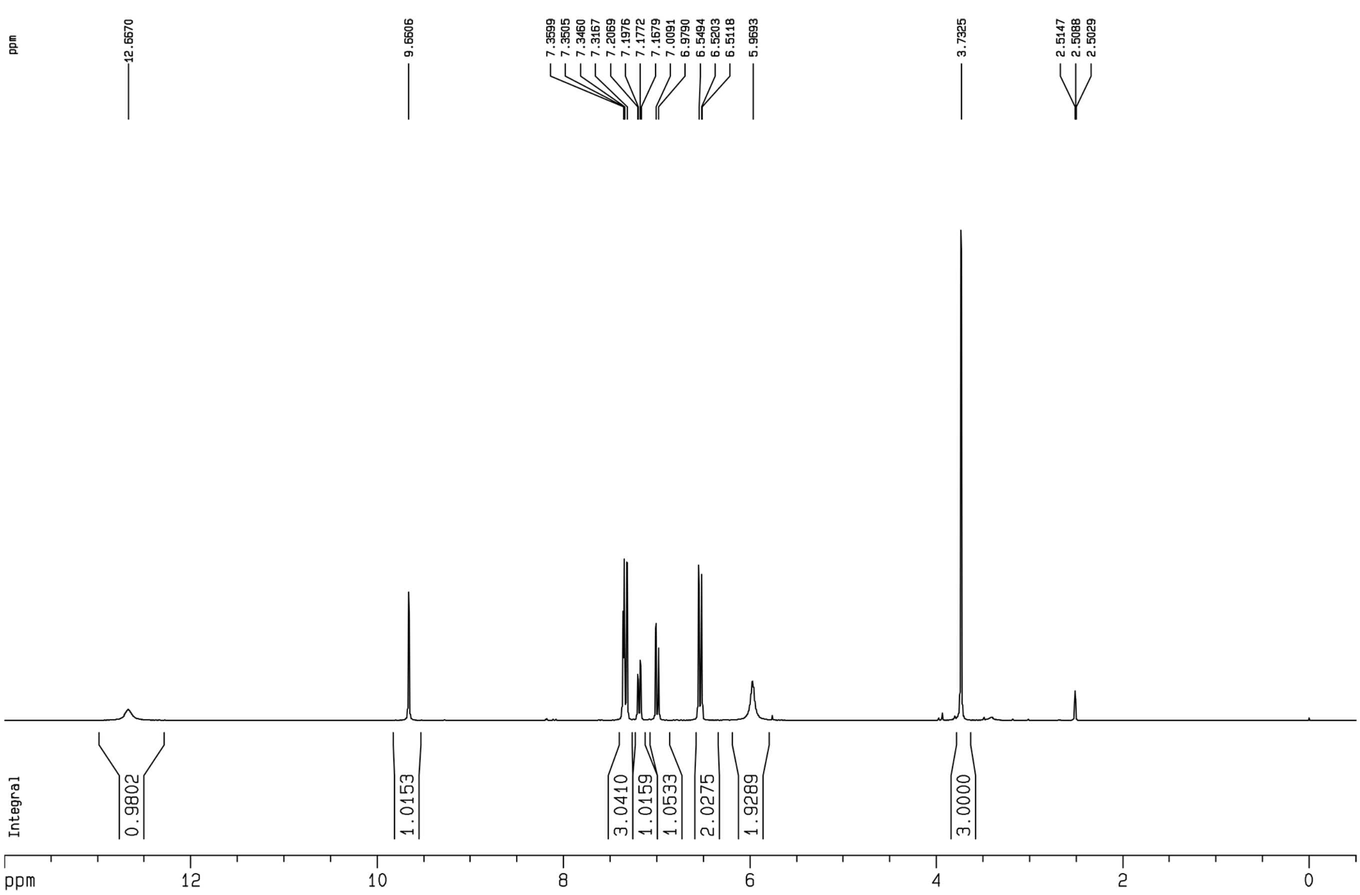
ppm
12.6670
9.6606
7.3599
7.3505
7.3460
7.3167
7.2069
7.1976
7.1772
7.1679
7.0091
6.9790
6.5494
6.5203
6.5118
5.9693
3.7325
2.5147
2.5088
2.5029
Integral
0.9802
1.0153
3.0410
1.0159
1.0533
2.0275
1.9289
3.0000
ppm
12
10
8
6
4
2
0

Figure S1.

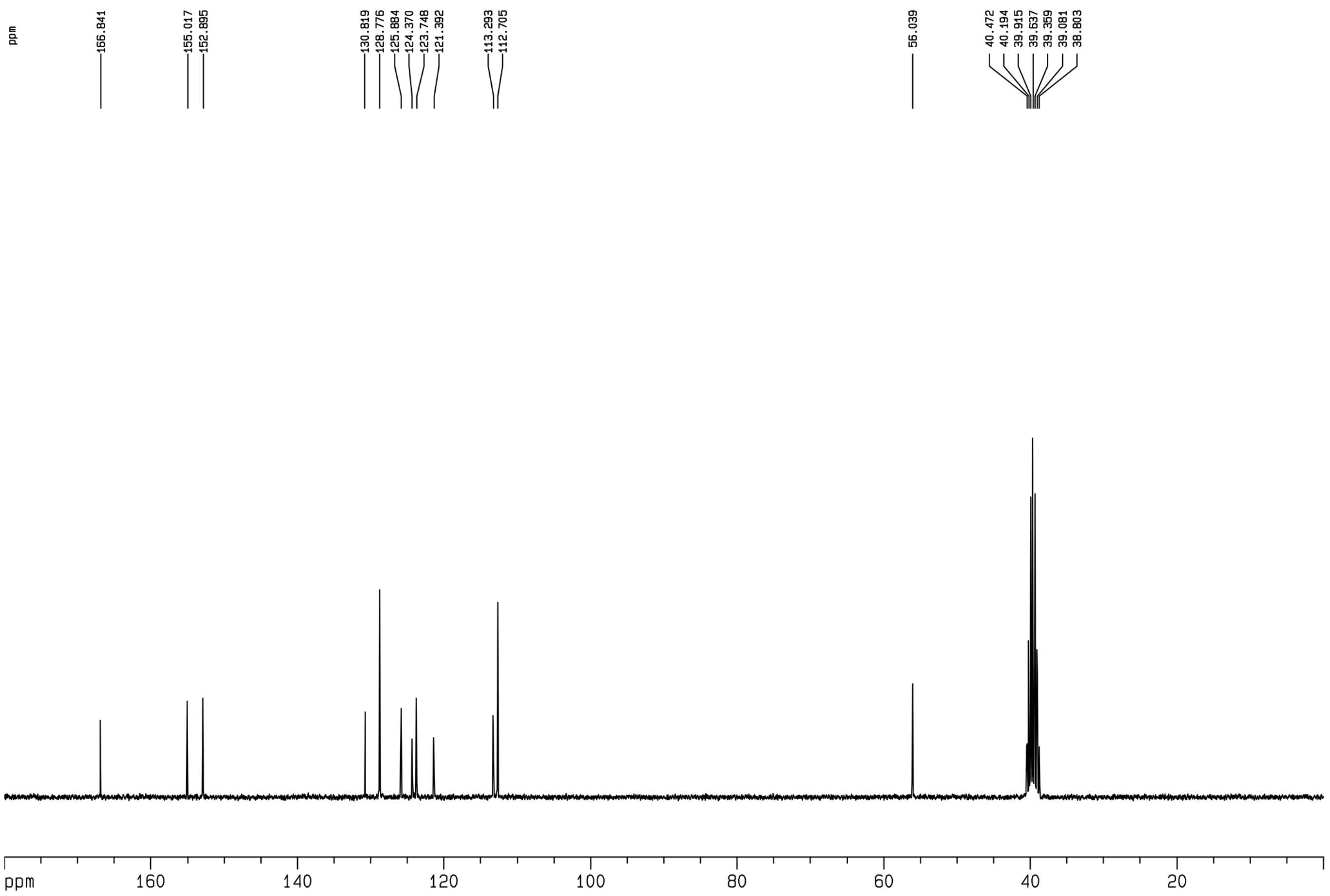
ppm
166.841
155.017
152.895
130.819
128.776
125.884
124.370
123.748
121.392
113.293
112.705
56.039
40.472
40.194
39.915
39.637
39.359
39.081
38.803
ppm
160
140
120
100
80
60
40
20

Figure S2.

References

1. Wang, Z.; Li, Y.; Liang, X.; Zhang, S.; Shi, W.; Shen, J. Forcing immunoassay for sulfonamides to higher sensitivity and broader detection spectrum by site heterologous hapten inducing affinity improvement. *Anal. Methods* **2013**, *5*, 6990–7000. doi: 10.1039/C3AY40864G
2. Ermolenko, D. N.; Eremin, S. A.; Mart'ianov, A. A.; Zherdev, A. V.; Dzantiev, B. B. A new generic enzyme immunoassay for sulfonamides. *Anal. Lett.* **2007**, *40*, 1047–1062. doi: 10.1080/00032710701296945
3. Franek, M.; Diblikova, I.; Cernoch, I.; Vass, M.; Hruska, K. Broad-specificity immunoassays for sulfonamide detection: Immunochemical strategy for generic antibodies and competitors. *Anal. Chem.* **2006**, *78*, 1559–1567. doi: 10.1021/ac0514422
4. Haasnoot, W.; Pre, J. D.; Cazemier, G.; Kemmers-Voncken, A.; Verheijen, R.; Jansen, B. J. M. Monoclonal antibodies against a sulfathiazole derivative for the immunochemical detection of sulfonamides. *Food Agric. Immunol.* **2000**, *12*, 127–138. doi: 10.1080/095401000404076
5. Zhang, H.; Duan, Z.; Wang, L.; Zhang, Y.; Wang, S. Hapten synthesis and development of polyclonal antibody-based multi-sulfonamide immunoassays. *J. Agric. Food Chem.* **2006**, *54*, 4499–4505. doi: 10.1021/jf060868u
6. Muldoon, M. T.; Font, I. A.; Beier, R. C.; Holtzapple, C. K.; Young, C. R.; Stanker, L. H. Development of a cross-reactive monoclonal antibody to sulfonamide antibiotics: Evidence for structural conformation-selective hapten recognition. *Food Agric. Immunol.* **1999**, *11*, 117–134. doi: 10.1080/09540109999807
7. Cliquet, P.; Cox, E.; Haasnoot, W.; Schacht, E.; Goddeeris, B. M. Generation of group-specific antibodies against sulfonamides. *J. Agric. Food Chem.* **2003**, *51*, 5835–5842. doi: 10.1021/jf034316c
8. Zhou, Q.; Peng, D.; Wang, Y.; Pan, Y.; Wan, D.; Zhang, X.; Yuan, Z. A novel hapten and monoclonal-based enzyme-linked immunosorbent assay for sulfonamides in edible animal tissues. *Food Chem.* **2014**, *154*, 52–62. doi: 10.1016/j.foodchem.2014.01.016
9. Korpimäki, T.; Hagren, V.; Brockmann, E.-C.; Tuomola, M. Generic lanthanide fluoroimmunoassay for the simultaneous screening of 18 sulfonamides using an engineered antibody. *Anal. Chem*. **2004**, *76*, 3091–3098. doi: 10.1021/ac049823n
10. Chen, Y.; Liu, L.; Xu, L.; Song, S.; Kuang, H.; Cui, G.; Xu, C. Gold immunochromatographic sensor for the rapid detection of twenty-six sulfonamides in foods. *Nano Res.* **2017**, *10*, 2833–2844. doi: 10.1007/s12274-017-1490-x
